# Neonatal Intensive Care Unit Evacuation and Care During a Natural Disaster: The Experience of Cyclone Idai in Beira, Mozambique

**DOI:** 10.3389/fped.2020.584281

**Published:** 2020-10-22

**Authors:** Serena Calgaro, Martina Borellini, Amir Hussein Abubacar Seni, Maria Concetta Tirzi, Antonio Marcos Dias Gimo, Bonifacio Rodriguez Cebola, Giovanni Putoto, Daniele Trevisanuto

**Affiliations:** ^1^Department of Woman's and Child's Health, University of Padova, Padova, Italy; ^2^Doctors With Africa CUAMM, Padova, Italy; ^3^Department of Pediatrics, Central Hospital of Beira, Beira, Mozambique

**Keywords:** NICU, neonatology, cyclone, natural disaster, Mozambique

## Abstract

Global warming has increased the frequency of natural disasters, such as cyclones. Mozambique is considered one of the most vulnerable countries to extreme weather events. Natural disasters particularly affect vulnerable people, including preterm and critical ill infants of Neonatal Intensive Care Units (NICUs). Literature on NICU evacuations in the case of a natural disaster has been reported in high-resource settings, but it is lacking in low-resource settings. On the 14th of March 2019, a tropical cyclone (Idai) hit Mozambique. This report is a descriptive analysis of the experience of the NICU evacuation and care during and after cyclone Idai at Beira Central Hospital, Beira, Mozambique.

## Introduction

During natural disasters Neonatal Intensive Care Units (NICU) patients are potentially the most vulnerable in a hospital structure. Despite this fact, literature on NICU evacuations remains very limited dealing with the experiences in high-resource settings (1–4). Barkemeyer published a descriptive report of NICU evacuation during Hurricane Katrina at the University Hospital of New Orleans in 2006 (1). After 5 years, he analyzed how the lessons learned from that experience were implemented, concluding that health care system preparation is the key component for facing extreme events (2). The presence of an evacuation plan allowed safe NICU evacuation in New York during Hurricane Sandy in 2012 (3). In 2017 Iwata et al. reported the first experience of a NICU evacuation after an earthquake in Japan, highlighting the urgency to develop a specific emergency plan for vulnerable infants (4).

Experience with NICU evacuations during natural disasters in low-resource settings, remains a knowledge gap.

On March 14, 2019, category 4 cyclone Idai struck Mozambique, Malawi, and Zimbabwe affecting 3 million people. In Mozambique alone, Idai killed at least 602 people, injured 1,641 and left an estimated 1.85 million people in need (5). In Beira, Mozambique, the cyclone and subsequent flooding severely damaged infrastructure and roads leaving the population isolated without electricity or communication for many days. All 17 health centers and the hospital were affected (3).

We report the events and the lessons learned from our experience at Beira Central Hospital in order to inform best practices in neonatal emergency care during future disasters.

### Hospital and NICU Background

The Central Hospital of Beira is a 1,040 bed referral hospital, located at 200 m from the coast. Every year, CHB records about 6,000 births. The hospital has a neonatal intensive care unit (NICU), the second biggest in Mozambique, service of reference for three provinces. Neonates aged 1–28 days of life are admitted to the NICU, with the lower level of viability around 28 weeks' gestation and/or birth weight around 900 g. Survival of extremely low birth weight infants is very rare. At CHB, structured plans for evacuation in case of emergency situations (i.e., natural disasters or fire) were not available.

In 2018, 2,176 newborns were admitted to the NICU, 49% of them were <2,500 g. The NICU is staffed by a local medical team of two residents and two general doctors, directed by a pediatrician from Cuba. A neonatologist and a pediatric fellow of Doctors with Africa CUAMM (an Italian non-governmental organization) continuously support the staff. Twenty-six local nurses and six local health workers complete the team. The neonatal ward is located on the 1st floor of the main building, near the Gynecology and Obstetrics Unit. It is organized in two sub-units: a 14 beds NICU and a 19 beds Kangaroo Mother Care (KMC) Unit. In the NICU there were seven incubators and six infant warmers, even if the bed capacity was often exceeded. The NICU offers the possibility of non-continuous monitoring of vital signs, respiratory assistance limited to non-invasive support (oxygen via nasal cannula, bubble continuous positive airway pressure - CPAP), intravenous hydration in peristaltic and syringe pump (even if not in sufficient number for all patients), umbilical and peripheral venous catheterization, phototherapy, enteral nutrition with gavage. A portable ultrasound machine is also available. There is no possibility to offer parenteral nutrition, invasive ventilation or therapeutic hypothermia.

### Pre-landfall

The population of Beira was alerted about 1 week before the cyclone's landfall, but no emergency plans were specifically organized. On March 14th, there were 13 sick infants in the NICU and 17 stable infants in the adjacent Kangaroo Mother Care (KMC) unit. One patient was receiving respiratory support with bubble CPAP, and other five were treated with supplemental oxygen.

The outer bands of the cyclone arrived on the morning, many hours before the Idai's eye impact. Because of logistic difficulties to reach the hospital, the nurse staff was reduced. Finally, that night the staff was composed by a doctor and a health care worker, as usual, but only by one nurse instead of three. None of them had previously received training on management of emergency situations or evacuation plans.

### Landfall

**Figure 1:**
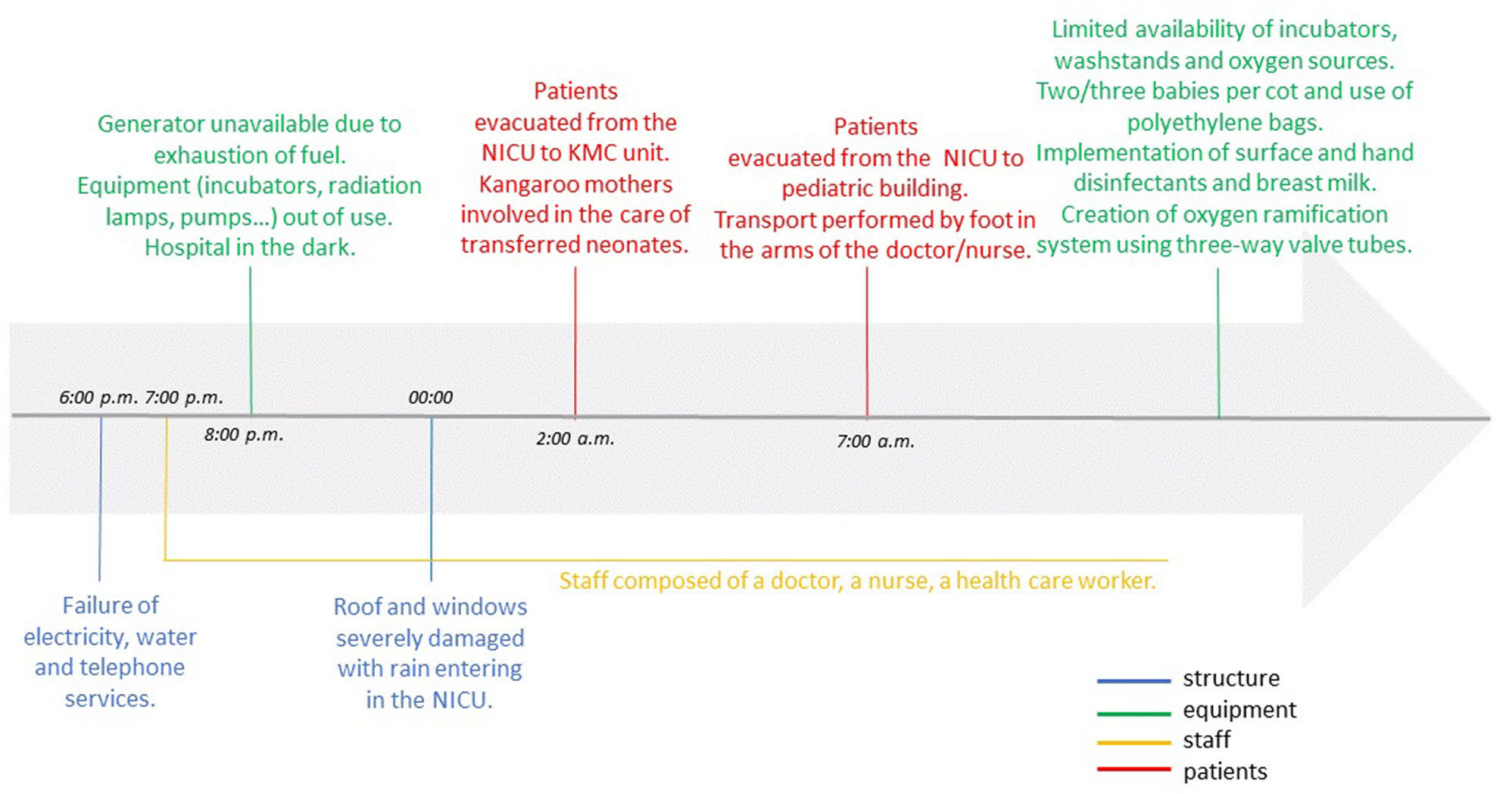
Time line of events and interventions.

Time line of events and interventions are reported in Figure 1.

#### 6:00 p.m.−0:00

In the afternoon of March 14th, Idai's eye made landfall on Mozambique's coast with winds that reached 121 miles/h. Shortly afterward, electrical power, water and telephone services failed. A generator briefly provided electricity but it was damaged by water. While functioning, the staff used their mobile phones for light and communication. The lack of electricity signified that incubators and radiation lamps usually used to keep warm the newborns didn't work; moreover, infusion and syringe pumps were out of action.

#### 00:00–2:00 a.m.

**Figure 2:**
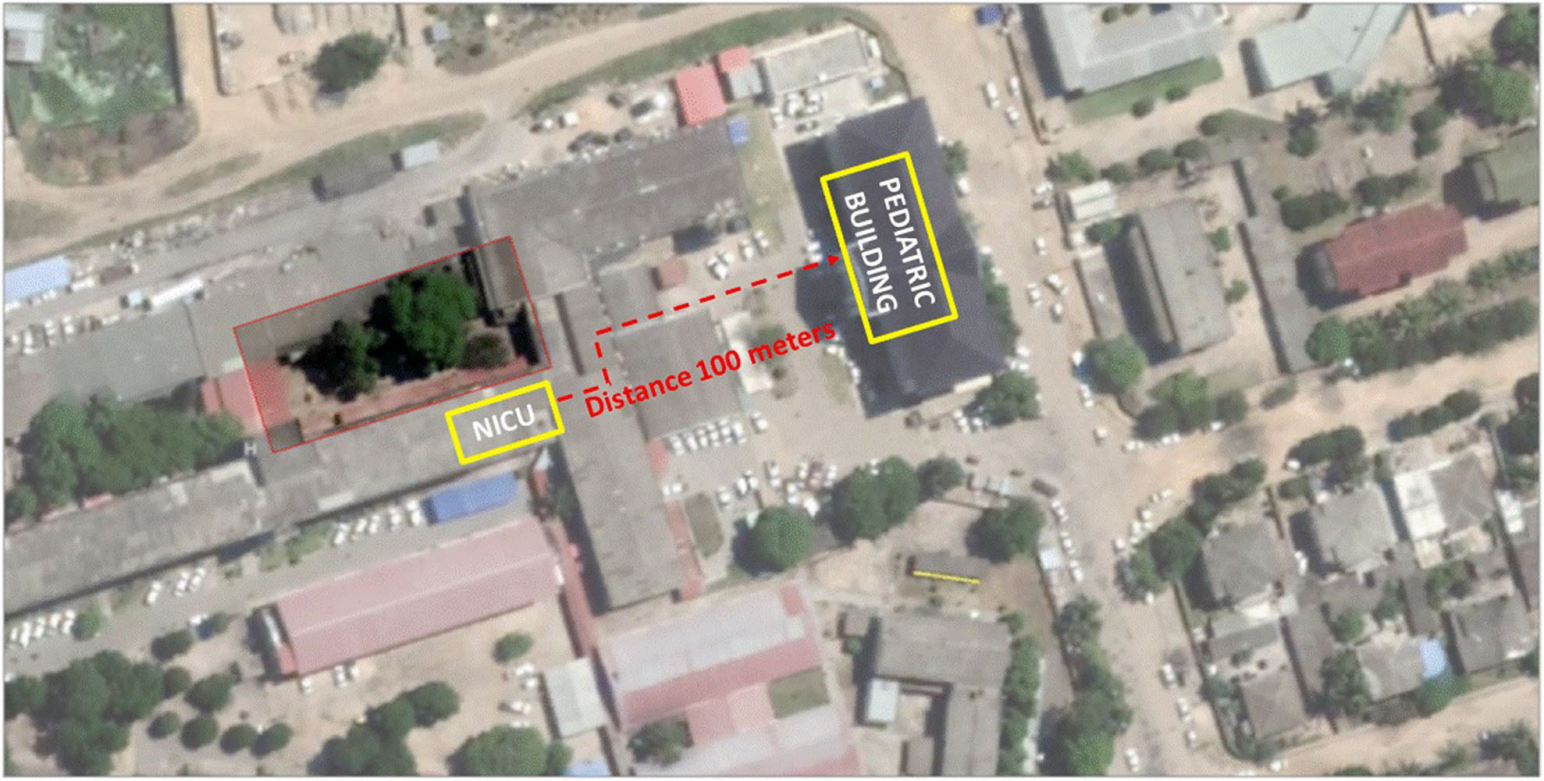
Layout of the original and “new” NICU at the CHB hospital.

Later that evening, a portion of the roof and windows were torn apart and rain entered the NICU (Figure 2). Neonates who were feeding with formula milk, didn't receive it during the night until 12:00 of the following day. Fortunately, all the NICU were receiving intravenous hydration and the infusions were administered without electric pumps.

At 2:00 a.m., the NICU patients were moved to the KMC unit where mothers helped care for the additional babies by wrapping them in dry sheets. Only one oxygen source was available and no cardiorespiratory monitors. Unfortunately, during the evacuation, a preterm suffered severe hypothermia. This likely contributed to his death on 15th March morning.

#### 2:00–7:00 a.m.

Fortunately, the area of KMC was not excessively damaged, except for some broken windows. The patients remained stable in this area, receiving only intravenous hydration and being kept warm with dry cloths, waiting for the improvement of the weather conditions. At 4:00 a.m. the cyclone's strength started to shrink, and subsided at 6:00 a.m.

#### 7:00–12:00 a.m.

**Figure 3:**
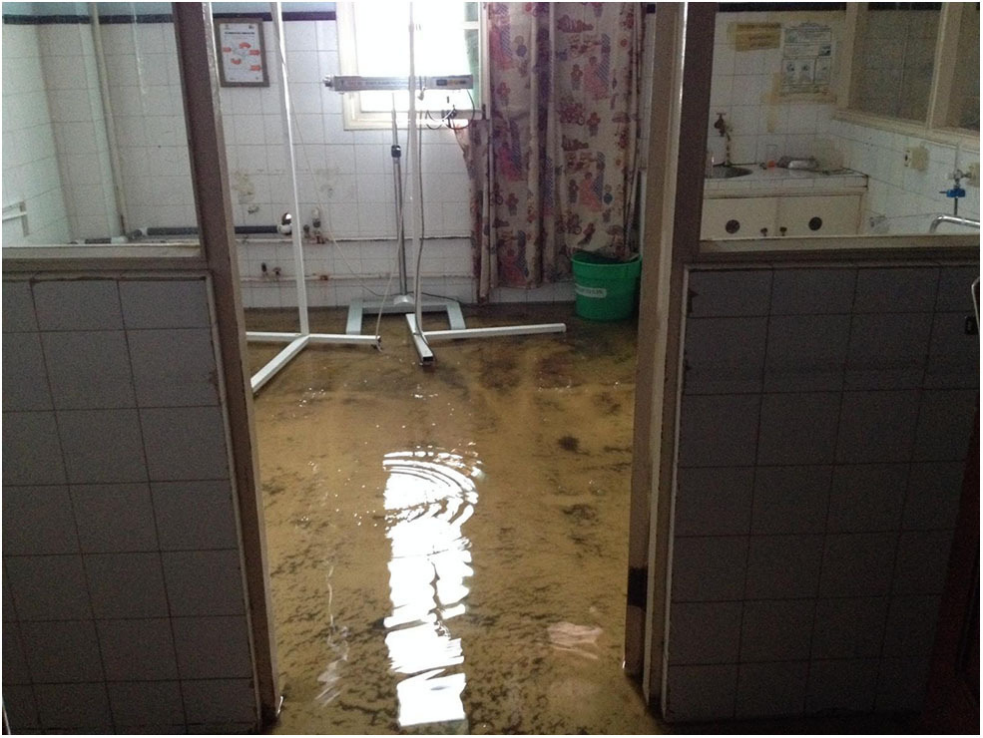
The neonatal intensive care unit the day after the cyclone.

As the NICU was severely damaged, two pediatric intensive care unit (PICU) rooms were adapted to care for NICU patients in an adjacent building (Figure 3). The evacuation was coordinated and performed by the doctor and the nurse who were on duty that night.

Babies were carried to the PICU in providers' arms because transport incubators were not available. Current medical records were rescued, but the NICU document archive was destroyed. Lacking communication equipment, the staff didn't know about the condition of their own families or when relief would arrive. In the NICU, a nurse had to work for 26 h continuously. Medical shift came at 12:00. Unfortunately, the number of kangaroo beds was considerably reduced, from 20 to 8.

### Recovery

The majority of hospital departments were severely damaged. In the “new” NICU, the staff faced challenges due to limited space, oxygen, and supplies for hygiene and thermoregulation. It was necessary to place more than one baby in each bed. Because there were only two oxygen sources, an improvised distribution system was created using multiple “Y” -connectors and tubing so each source could provide oxygen to 3–5 infants. As incubators were damaged, radiant lamps were used to prevent hypothermia and polyethylene bags were used for preterm infants. There was no washstand, but surface and hand disinfectants were used when possible. For milk preparation, water was boiled and additional water filters were used. The use of breast milk in the NICU and kangaroo care were further supported. Locally, flooding resulted in a significant increase in the incidence of malaria and a cholera outbreak (6), and one infant with cholera was isolated and treated. For a long time after the cyclone Idai, medical and nurse staff suffered human and material losses. Nevertheless, demonstrating resilience and resourcefulness, the local NICU staff never stopped to offer their service to the patients and their mothers.

The humanitarian response in Beira was coordinated by the Government and was supported by United Nations Agencies, international NGOs and Red Coss. In particular, an advanced medical post of the Italian Civil Protection was built within the CHB. In the emergency phase, the NICU was supported by an integrated collaboration between CUAMM doctors, Italian Red Cross staff and the portuguese NGO HealthforMoz with international staff, drugs and equipment supply and emergency strategies.

### Current Situation

The NICU is going to be rebuilt. This is the opportunity to adapt the NICU infrastructure and equipment to emergency situations, considering the high risk of extreme weather events in Mozambique. A preparedness evacuation plan is going to be created at the CHB.

## Discussion and Lessons Learned

This is the first report describing a NICU evacuation after a natural disaster in Africa. Many of the challenges faced by the staff in Beira have not been reported because of the unique features of a low resource setting. Following debriefing with the staff, we have identified lessons learned from our experience that can be generalized to NICUs in other resource limited settings (Table 1).

**Table 1:** Problems and possible solutions.

**Issue**	**Problem**	**Solution**
Prevention	Although the population was pre-alerted, the cyclone's impact was underestimated	Implementation of a local emergency plan
Triage	Patients were evacuated without a specific order of priority	Staff education on the triage to adopt in local emergency situations
Oxygen supply	Lack of oxygen sources	System with multiple “Y” -connectors and tubing to provide oxygen to more babies from an oxygen source
Thermal control	Loss of incubators and infant warmers (one preterm infant had severe hypothermia. This likely contributed to his death)	Use of polyethylene bags Implementation of kangaroo mother care Education on thermoregulation of preterm infants Preparation of a new medical chart with more emphasis on temperature
Hygiene	No washstand	Implementation of disinfectant use kangaroo mother care
Nutrition	Milk preparation	Water filters Use of fresh breast milk in the NICU Kangaroo mother care, breastfeeding
Electricity	Due to electrical blackout, communication in and outside hospital were interrupted; staff worked in the dark, unaware of their relatives' conditions at home; medical devices stopped to work	Mobile phones were used to have light and for communication in and outside hospital, but an independent hospital generator should be implemented
Patient records	NICU archive was destroyed and patient records were definitively lost	Opportunity to create an electronic hospital archive that can be saved in an external electronic store
Local and international aids	Local and international coordination of health issues during post Idai phase has been challenging	A strict cooperation between local and international institutions, including a defined leadership, is mandatory to avoid waste of human and economic resources

### Preparation

Preparation has been identified as the key component for limiting damages during disasters (7). Although the population was alerted, the cyclone's impact was underestimated and there was no pre-existing disaster preparedness plan. In low-resource settings, the lack of effective risk management systems, technology and infrastructure make adequate preparation challenging. Our experienced emphasized the importance of local NICU staff developing disaster preparedness plans that include triage priorities, contingency planning and evacuation plans. However, a written plan does not ensure preparedness. The effort, courage and improvisation exhibited by the staff in Beira was commendable and inspiring; however, training on the response to emergency situations must become a part of the basic education for all healthcare staff and disaster preparedness must become a part of the hospital culture. It is critically important for the hospital leadership and staff to train and regularly practice with disaster drills so existing plans can be rapidly implemented. Moreover, we believe that NICU staff must develop a sense of ownership in the plans and regularly update them to ensure they remain useful.

### Resource Allocation

The disaster response has to consider available resources and must be tailored to the country's health service. In settings where emergency response facilities and technology may not be available, the most effective resource is the community. During a crisis, the greatest impact may be achieved by employing local resources and simple remedies rather than relying on the rapid deployment of international aid (5). In our experience, the dedication of the local staff and the involvement of mothers providing kangaroo care addressed key challenges during the acute emergency and post-emergency phases. After the disaster, the risk of contaminated water and subsequent diarrheal disease is high. For this reason, the use of formula milk should be reduced to a minimum and breastfeeding should be encouraged. Kangaroo mother care can help with thermoregulation, provision of safe nutrition, infection prevention and reducing overcrowding by allowing early discharge. We believe that early and expanded implementation of kangaroo care is one of the key strategies for managing disasters in NICUs with limited resources. In addition, adequate supplies to support hand hygiene, provide safe drinking water and food for staff and lactating mothers, culturally appropriate sanitation, and medications to treat malaria, cholera and continue HIV prophylaxis must be considered. Other simple strategies, such as portable oxygen with an oxygen distribution system, use of independent battery operated communication devices, and the use of polyethylene bags for neonatal thermoregulation should be considered.

### International Aid

Finally, the governmental response to Idai was supported by international aid. The coordination of local and international aid is challenging, but fundamental to managing complex problems such as infectious disease outbreaks and rebuilding the health infrastructure. In Mozambique, international organizations ensured adequate staffing, provided medications and allowed the possibility of constructing a new NICU. Successful cooperation between local and international institutions is mandatory to avoid duplication of aid in some areas while leaving others uncovered.

## Conclusions

This report is a descriptive analysis of NICU evacuation during a natural disaster in Africa. Consistently with high-resource setting experiences, we reported that preparation, situation awareness, communication, clear coordination structure, flexibility, and adaptability in utilizing the available resources and staff dedication are of pivotal importance to ensure continuous care for critically ill neonates during a natural disaster in a low-resource setting.

## Data Availability Statement

The original contributions presented in the study are included in the article/supplementary material, further inquiries can be directed to the corresponding author/s.

## Author Contributions

SC and MB performed the literature review, collected the data, contributed to data interpretation, drafted the initial manuscript, and critically reviewed the manuscript. AS, MT, AG, and BC contributed to the collection and analysis of data, contributed to data interpretation and reviewed, and critically reviewed the manuscript. GP and DT conceptualized the study, contributed to data interpretation, writing of the manuscript, and critically reviewed the manuscript. All authors approved the final manuscript as submitted and agree to be accountable for all aspects of the work.

## Conflict of Interest

The authors declare that the research was conducted in the absence of any commercial or financial relationships that could be construed as a potential conflict of interest.
